# Lasting impairments following transient ischemic attack and minor stroke: a systematic review protocol

**DOI:** 10.3389/fneur.2023.1177309

**Published:** 2023-05-12

**Authors:** Birgitte Hede Ebbesen, Boris Modrau, Eirini Kontou, Emma Finch, Gary Crowfoot, Jennifer Crow, Neil Heron, Tenelle Hodson, Conni Skrubbeltrang, Grace Turner

**Affiliations:** ^1^Department of Physiotherapy and Occupational Therapy, Aalborg University Hospital, Aalborg, Denmark; ^2^Department of Health Science and Technology, Aalborg University, Aalborg, Denmark; ^3^Department of Neurology, Aalborg University Hospital, Aalborg University, Aalborg, Denmark; ^4^Mental Health and Clinical Neurosciences, School of Medicine, University of Nottingham, Nottingham, United Kingdom; ^5^Institute of Mental Health, Nottinghamshire Healthcare NHS Foundation Trust, Nottingham, United Kingdom; ^6^Research and Innovation, West Moreton Health, Ipswich, QLD, Australia; ^7^Speech Pathology Department, Princess Alexandra Hospital, Woolloongabba, QLD, Australia; ^8^School of Health and Rehabilitation Sciences, The University of Queensland, Brisbane, QLD, Australia; ^9^School of Nursing and Midwifery, College of Health, Medicine and Wellbeing, University of Newcastle, Callaghan, NSW, Australia; ^10^Department of Brain Sciences, Imperial College London, Imperial College Healthcare NHS Trust, London, United Kingdom; ^11^Centre for Public Health, Queen’s University Belfast, Belfast, Ireland; ^12^School of Medicine, Keele University, Staffordshire, United Kingdom; ^13^School of Health Sciences and Social Work and The Hopkins Centre, Menzies Health Institute Queensland, Griffith University, Nathan, QLD, Australia; ^14^Medical Library, Aalborg University Hospital, Aalborg, Denmark; ^15^Institute of Applied Health Research, University of Birmingham, Birmingham, United Kingdom

**Keywords:** transient ischemic attack, TIA, minor stroke, mild stroke, impairments, systematic review, protocol

## Abstract

**Introduction:**

The focus on medical management and secondary prevention following Transient Ischemic Attack (TIA) and minor stroke is well-established. Evidence is emerging that people with TIA and minor stroke can experience lasting impairments as fatigue, depression, anxiety, cognitive impairment, and communication difficulties. These impairments are often underrecognized and inconsistently treated. Research in this area is developing rapidly and an updated systematic review is required to evaluate new evidence as it emerges. This living systematic review aims to describe the prevalence of lasting impairments and how they affect the lives of people with TIA and minor stroke. Furthermore, we will explore whether there are differences in impairments experienced by people with TIA compared to minor stroke.

**Methods:**

Systematic searches of PubMed, EMBASE, CINAHL, PsycINFO, Cochrane Libraries will be undertaken. The protocol will follow the Cochrane living systematic review guideline with an update annually. A team of interdisciplinary reviewers will independently screen search results, identify relevant studies based on the defined criteria, conduct quality assessments, and extract data. This systematic review will include quantitative studies on people with TIA and/or minor stroke that report on outcomes in relation to fatigue, cognitive and communication impairments, depression, anxiety, quality of life, return to work/education, or social participation. Where possible, findings will be grouped for TIA and minor stroke and collated according to the time that follow-up occurred (short-term < 3 months, medium-term 3–12 months, and long-term > 12 months). Sub-group analysis on TIA and minor stroke will be performed based on results from the included studies. Data from individual studies will be pooled to perform meta-analysis where possible. Reporting will follow the Preferred Reporting Items for Systematic review and Meta-Analysis Protocol (PRISMA-P) guideline.

**Perspectives:**

This living systematic review will collate the latest knowledge on lasting impairments and how these affect the lives of people with TIA and minor stroke. It will seek to guide and support future research on impairments emphasizing distinctions between TIA and minor stroke. Finally, this evidence will allow healthcare professionals to improve follow-up care for people with TIA and minor stroke by supporting them to identify and address lasting impairments.

## 1. Introduction

Evidence of lasting impairments following transient ischemic attack (TIA) and minor stroke has emerged over the last two decades. These impairments include fatigue, cognitive difficulties, depression, and anxiety (1–6). Some people with TIA experience lasting impairments despite a diagnosis of TIA where definitions state that symptoms should resolve completely within 24 h. The lack of consensus on how TIA should be defined is problematic. The time-based definition states that symptoms should resolve within 24 h (7), whereas the tissue-based definition is not limited by time, but based on the absence of an acute infarction on imaging (8). Definitions of minor stroke also vary (9). The extent of disability can be used to define whether a stroke is minor or not. However, several different measures are used to identify the level of disability. The National Institute of Health Stroke Scale (NIHSS) is commonly used to define minor stroke, with a score of 5 or less being considered a minor stroke (10). Yet, variability exists with cut-offs within the literature (11, 12). Another way of defining minor stroke is to broadly describe it as an ischemic event resulting in minor neurological impairment or disability (13).

Persons with TIA and minor stroke are often quickly discharged from acute health services and have limited follow up through services such as outpatient clinics. The importance of secondary prevention to avoid recurrent events is well established and clearly outlined in international guidelines (7). In contrast, there are no guidelines that specifically address how best to support people with lasting impairments following TIA or minor stroke to resume usual daily activities.

Two quantitative systematic reviews (1, 2) on lasting impairments following TIA and/or minor stroke have been published. Moran et al. found high levels of cognitive impairment and depression following TIA or minor stroke (2). Cognitive impairments in TIA or minor stroke were reported in 13 of 31 studies but variations were reported in the measurement tools and prevalence (17% to 54%) (2). A limited number of studies reported evidence of anxiety (14, 15) and fatigue in both TIA and minor stroke (16–18). In their systematic review, Rooij et al. found high variation in reported rates of cognitive impairment following TIA and concluded that mild cognitive impairment is present in more than one third of people who experience TIA (1). The findings from these reviews provide evidence of lasting impacts related to cognition, fatigue, and psychological well-being. However, an updated review in this rapidly evolving field of research that more comprehensively captures the lasting impacts of TIA and minor stroke is warranted. For instance, recent research has published and solidified findings around emotional impairments following TIA and minor stroke (4, 6, 18), fatigue following minor stroke (17), and impairments in communication, quality of life, social participation and return to work following minor stroke (9). Also the qualitative research describes life-altering experiences following TIA or minor stroke with changes in multiple life-domains, which were overlooked and undetected by healthcare professionals (19).

An updated, living systematic review that evaluates new evidence, as it emerges, is required to capture the rapidly growing body of research in this area. Thus, we aim to describe a protocol for a living systematic review on lasting impairments following TIA or minor stroke. The review will be an updated version of the systematic review by Moran et al. (2). Furthermore, it will be an extended version by including additional impairments identified in the recent literature. The review will guide future research and support healthcare professionals to deliver evidence-based care.

## 2. Materials and methods

This protocol describes a living systematic review which is defined as “a systematic review that is continually updated, incorporating relevant new evidence as it becomes available” (20, p. 6). The Cochrane guidelines for a living systematic review will be followed (20). Reasons for making the systematic review living are the rapidly growing research in this area and the need for timely updates on the available evidence. There is still uncertainty in the existing body of literature on lasting impairments following TIA or minor stroke and a living systematic review will allow to regularly incorporate new and relevant research. The reporting of the protocol also follows the Preferred Reporting Items for Systematic review and Meta-Analysis Protocol (PRISMA-P) guideline.

### 2.1. Eligibility for study inclusion

#### 2.1.1. Participants

Participants in the included studies must be 18 years or older. If younger participants are included 90% of the total sample must be over 18 years of age (2), since this review aims to describe the adults experiences which can differ from that of children. All studies that state inclusion of participants with TIA, minor stroke, mild stroke, non-disabling stroke, or reversible stroke will be included. All stated definitions on TIA or minor stroke will be eligible and so no limitations on neuroimaging, outcome scores or similar will be used. This is to ensure the inclusion of relevant studies despite the inconsistencies in TIA and minor stroke definitions and terminology used in the published research literature. Studies including participants who have received reperfusion therapy is also eligible for inclusion as long as they are diagnosed minor stroke at discharge. Studies including other types of stroke or neurological disorders will only be included if it is possible to extract outcomes and data separately for TIA and/or minor stroke. Participants with described “silent” strokes are not included in the review, since it is not possible to determine symptoms and impairment in relation to ischemic lesions occurred back in time.

#### 2.1.2. Design and outcome

All study designs with quantitative outcomes are eligible for inclusion except singe-case designs, reviews, or expert opinions. Interventional studies where the intervention comprises of interventions post discharge on participant with TIA or minor stroke will be included if it is possible to extract data on participants who only receive usual care, since this is not a review of the effects of interventions.

Studies reporting on the following outcomes will be included: fatigue, cognitive and communication impairments, depression, anxiety, quality of life, return to work/education or social participation. There will be no restrictions on the length of follow-up or time since TIA or minor stroke.

### 2.2. Information sources

Systematic searches will be conducted in PubMed (National Library of Medicine), Embase (Elsevier), CINAHL with Full Text (Ebsco), PsycInfo (APA PsycNet), and Cochrane Library (Wiley).

References from included studies will be scanned and tracked through the cited reference search in Scopus (Elsevier) to secure the inclusion of all relevant studies.

### 2.3. Search strategy

A comprehensive search strategy has been developed using both controlled vocabulary terms (i.e., MeSH terms) adapted for each database and natural language words for TIA and a variety of words covering different impairments. Search terms have been established by initial searches in all included databases and builds upon the strategy developed in a previously published review by Moran et al. (2). The search strategy was created in cooperation with specialist medical librarians.

To combine and expand upon the previous review Moran et al. (2) the searches will be limited to publication years from 2013. There will be no restrictions to language.

A detailed draft of search strategies for all databases can be found in Additional File 1.

Search strategies have been peer reviewed by a specialist medical librarian outside the author group and will be run annually to identify new relevant studies. The search strategy will also be updated every year to ensure that it reflects the relevant terminology in the research area and the included search databases.

### 2.4. Data management and selection process

Records will be imported to, and the review process handled in, the systematic review software Covidence (21).

Two interdisciplinary reviewers will independently screen title and abstracts to include full text articles. Any disagreements will be resolved by consensus. Interdisciplinary reviewers will independently screen the retrieved full text articles for the defined inclusion criteria.

The described procedure for data management and selection of full text articles will be applied every year for the updated searches.

### 2.5. Quality assessment

Quality assessment will be assessed by two independent reviewers. Based on the study design of the included studies, appropriate checklists from the Joanna Briggs Institute will be used to asses quality and risk of bias (22). When one or more from the author group are listed as authors of included studies, other members of the author group will perform the quality assessment and data extraction.

The described procedures for quality assessments and risk of bias will be applied every year for new relevant studies identified in the updated searches.

### 2.6. Data extraction and analysis of results

Data will be extracted in duplicates by two independent reviewers and compared to reach consensus on the final data extraction. A data extraction form will be developed to extract data on bibliographic information, study design, population, risk of bias assessments, possible comparators, measurement tool and outcomes (both numerical results and effect estimates) (23). The data extraction will be handled in Covidence and Microsoft Excel/Research Electronic Data Capture (REDCap). In case of missing data the original investigators of the study will be contacted to obtain the relevant data. If the same study is published in multiple publications, the review authors will extract and combine any relevant data. If in doubt of which data to include, the original publication will be prioritized.

Results of lasting impairments will be categorized and summarized according to the domains of identified difficulties (depression, anxiety, fatigue, quality of life, communication, and cognitive impairments, return to work/education and social participation). Furthermore, results will be reported based on length of follow-up (short-term < 3 months, medium-term 3–12 months, and long-term > 12 months). If appropriate sub-group analysis on definitions of TIA and minor stroke will be performed based on the results and participant characteristics in the included studies.

The results for the individual outcomes will be pooled to conduct meta-analysis if studies demonstrate clinical and methodological homogeneity. Only studies that demonstrate moderate or low risk of bias will be included in meta-analysis. Homogeneity will be evaluated based on the comparability of study designs, results confidence intervals and risk of bias as well as statistically using Chi-square in conjunction with I^2^ statistics where I^2^ < 60% is considered low-moderate heterogeneity (23–25). If studies are considered homogeneous a random-effects model will be used to pool estimates (23–25). If deemed suitable meta-analysis will also be performed for the sub-group populations. Sensitivity analysis will be performed and these will be specified during the selection process as described by the Cochrane Handbook of Systematic Reviews (25).

A narrative synthesis of results will be provided when a meta-analysis is not appropriate due to heterogeneity.

### 2.7. Transitioning out of living mode

Whether or not the living systematic review should continue to be in living mode will be evaluated each year (20). This review will transition out of living mode if the described uncertainties in lasting impairments following TIA or minor stroke no longer exists or if no new research emerges that can impact the conclusions of the review (20). When the review transitions out of living mode, this will be described in the latest update.

## 3. Synthesis of results

Results will be presented in accordance with the described domains, length of follow-up and sub-group analysis. All included studies will be summarized in a table presenting study design, participant characteristics, sample size, outcomes, and measurement tools used.

When updates of the living systematic review have been performed, new studies will be presented in “What’s New” tables as proposed by Cochrane guidelines (20). A new publication of the systematic review with summarized results and meta-analysis will be conducted when the authors assess that new evidence alters the nature and understanding of impairments following TIA or minor stroke.

## 4. Discussion

Research in the area of lasting impairments following TIA and minor stroke is increasing. This highlights the need for an updated systematic review to collate the emerging evidence. New findings generated in this review will support improvements in knowledge and understanding in this area. By comprehensively understanding the global evidence base surrounding lasting impacts of TIA and minor stroke, it is anticipated that this review can help to inform the development of interventions that meet the needs of these populations. Furthermore, it will help identify research gaps that can be targeted in the future.

A challenge in research and clinical settings is the lack of consensus regarding the definition used for the diagnosis of TIA or minor stroke. Internationally, there are ongoing discussions on how to differentially diagnose TIA and minor stroke. This discrepancy in diagnosis can result in patients with the same symptom duration and imaging being diagnosed as TIA in some countries and minor stroke in others. This discrepancy was underlined by Easton and Johnston in their opinion statement “Time to Retire the Concept of Transient Ischemic Attack” (26). They state that TIA is minor stroke. They argue, that as the quality of imaging improves evidence of permanent tissue damage will be present in many of these transient events. Using the tissue-based definition of TIA (8) this will result in many of these patients being diagnosed with minor stroke instead of TIA. This discrepancy in definitions and inequality to access imaging also challenges sub-group analysis based on the presence of acute infarction, since undetected or unreported ischemic lesions might be present.

The different approaches used to diagnose TIA and minor stroke challenges the ability to compare cohorts between studies and countries. It is also a limitation to this systematic review since it is estimated that different definitions are used in the research literature. We have chosen a pragmatic, real world approach to this and include studies stating inclusion of people with TIA or minor stroke. This is anticipated to strengthen the clinical relevance of the review where sub-group analysis will provide more clarity on symptoms and differences between definitions of TIA and minor stroke.

In many countries eligibility to access post stroke pathways and services often relies on a diagnosis of stroke. People with a diagnosis of TIA can thus be denied access to the support they may require to manage lasting impairments that impact their quality of life, ability to return to work, and participate socially. The results from this living systematic review will provide updated knowledge on lasting impairments following TIA or minor stroke and, where impairments could differ between the two. This will guide future research and provide valuable information for healthcare professionals to inform improvements in patient care.

## Author contributions

GT established the author group. BH, BM, EK, EF, GC, JC, NH, TH, CS, and GT contributed to the idea and content of the protocol including search terms and eligibility criteria for included studies. BH made the first drafts for the protocol in cooperation with GC and JC. All authors contributed to the article and approved the submitted version.

## Funding

This work was supported by Aalborg University Hospital, Denmark for publication fees.

## Conflict of interest

The authors declare that the research was conducted in the absence of any commercial or financial relationships that could be construed as a potential conflict of interest.

## Publisher’s note

All claims expressed in this article are solely those of the authors and do not necessarily represent those of their affiliated organizations, or those of the publisher, the editors and the reviewers. Any product that may be evaluated in this article, or claim that may be made by its manufacturer, is not guaranteed or endorsed by the publisher.
