# Supplementary material for: Lasting impairments following transient ischemic attack and minor stroke: a systematic review protocol
Source: Front Neurol. 2023 May 12;14:1177309. doi: 10.3389/fneur.2023.1177309 (PMC10213239; doi:10.3389/fneur.2023.1177309)
Supplement: Supplementary file 1 [file Data_Sheet_1.pdf]

Additional file 1

Database	Results	Date
PubMed, National Library of Medicine	14,165	2023.02.15
Embase.com, Elsevier	10,517	2023.02.15
Cinahl with Full Text, Ebsco	1,248	2023.02.15
Cochrane Library	881	2023.02.15
APA PsycNet	547	2023.02.15

PubMed

Search number	Search Details	Results
5	("ischemic attack, transient"[MeSH Terms] OR (("minor"[Text Word] OR "mild"[Text Word] OR "mini"[Text Word] OR "non disabling"[Text Word] OR "nondisabling"[Text Word] OR "transient"[Text Word] OR "non severe*"[Text Word] OR "nonsevere*"[Text Word] OR "reversib*"[Text Word]) AND ("stroke"[Text Word] OR "ischemi*"[Text Word] OR "ischaemi*"[Text Word] OR "cerebral"[Text Word] OR "cerebro*"[Text Word] OR "CVA"[Text Word])) OR "TIA"[Text Word]) AND ("Cognition"[MeSH Terms] OR "Cognition Disorders"[MeSH Terms] OR "Fatigue"[MeSH Terms] OR "Anxiety"[MeSH Terms] OR "Depression"[MeSH Terms] OR "Depressive Disorder"[MeSH Terms] OR "Return to Work"[MeSH Terms] OR "Speech Disorders"[MeSH Terms] OR "Social Communication Disorder"[MeSH Terms] OR "Social Participation"[MeSH Terms] OR "Quality of Life"[MeSH Terms] OR "cogniti*"[Text Word] OR "Fatigue"[Text Word] OR "anxiet*"[Text Word] OR "depress*"[Text Word] OR "HRQOL"[Text Word] OR "Health Related Quality Of Life"[Text Word] OR "Life Quality"[Text Word] OR "Return to Work"[Text Word] OR "back to work"[Text Word] OR "memory"[Text Word] OR "speech"[Text Word] OR "aphasia"[Text Word] OR "communicat*"[Text Word] OR "social limit*"[Text Word] OR "social participat*"[Text Word] OR "social engage*"[Text Word] OR "Quality of Life"[Text Word] OR "impairment*"[Text Word]) AND 2013/01/01:3000/12/31[Date - Publication]	**14,165**
4	2013/01/01:3000/12/31[Date - Publication]	12,675,300
3	("ischemic attack, transient"[MeSH Terms] OR (("minor"[Text Word] OR "mild"[Text Word] OR "mini"[Text Word] OR "non disabling"[Text Word]	28,738

	OR "nondisabling"[Text Word] OR "transient"[Text Word] OR "non severe*"[Text Word] OR "nonsevere*"[Text Word] OR "reversib*"[Text Word]) AND ("stroke"[Text Word] OR "ischemi*"[Text Word] OR "ischaemi*"[Text Word] OR "cerebral"[Text Word] OR "cerebro*"[Text Word] OR "CVA"[Text Word])) OR "TIA"[Text Word]) AND ("Cognition"[MeSH Terms] OR "Cognition Disorders"[MeSH Terms] OR "Fatigue"[MeSH Terms] OR "Anxiety"[MeSH Terms] OR "Depression"[MeSH Terms] OR "Depressive Disorder"[MeSH Terms] OR "Return to Work"[MeSH Terms] OR "Speech Disorders"[MeSH Terms] OR "Social Communication Disorder"[MeSH Terms] OR "Social Participation"[MeSH Terms] OR "Quality of Life"[MeSH Terms] OR "cogniti*"[Text Word] OR "Fatigue"[Text Word] OR "anxiet*"[Text Word] OR "depress*"[Text Word] OR "HRQOL"[Text Word] OR "Health Related Quality Of Life"[Text Word] OR "Life Quality"[Text Word] OR "Return to Work"[Text Word] OR "back to work"[Text Word] OR "memory"[Text Word] OR "speech"[Text Word] OR "aphasia"[Text Word] OR "communicat*"[Text Word] OR "social limit*"[Text Word] OR "social participat*"[Text Word] OR "social engage*"[Text Word] OR "Quality of Life"[Text Word] OR "impairment*"[Text Word])	
2	"Cognition"[MeSH Terms] OR "Cognition Disorders"[MeSH Terms] OR "Fatigue"[MeSH Terms] OR "Anxiety"[MeSH Terms] OR "Depression"[MeSH Terms] OR "Depressive Disorder"[MeSH Terms] OR "Return to Work"[MeSH Terms] OR "Speech Disorders"[MeSH Terms] OR "Social Communication Disorder"[MeSH Terms] OR "Social Participation"[MeSH Terms] OR "Quality of Life"[MeSH Terms] OR "cogniti*"[Text Word] OR "Fatigue"[Text Word] OR "anxiet*"[Text Word] OR "depress*"[Text Word] OR "HRQOL"[Text Word] OR "Health Related Quality Of Life"[Text Word] OR "Life Quality"[Text Word] OR "Return to Work"[Text Word] OR "back to work"[Text Word] OR "memory"[Text Word] OR "speech"[Text Word] OR "aphasia"[Text Word] OR "communicat*"[Text Word] OR "social limit*"[Text Word] OR "social participat*"[Text Word] OR "social engage*"[Text Word] OR "Quality of Life"[Text Word] OR "impairment*"[Text Word]	2,755,141
1	"ischemic attack, transient"[MeSH Terms] OR (("minor"[Text Word] OR "mild"[Text Word] OR "mini"[Text Word] OR "non disabling"[Text Word] OR "nondisabling"[Text Word] OR "transient"[Text Word] OR "non severe*"[Text Word] OR "nonsevere*"[Text Word] OR "reversib*"[Text Word]) AND ("stroke"[Text Word] OR "ischemi*"[Text Word] OR "ischaemi*"[Text Word] OR "cerebral"[Text Word] OR "cerebro*"[Text Word] OR "CVA"[Text Word])) OR "TIA"[Text Word]	129,476

Embase

No.	Query	Results
#9	#4 AND #7 AND [2013-2023]/py	**10517**
#8	#4 AND #7	15905
#7	#5 OR #6	5532268
#6	cogniti*:ti,ab,kw OR fatigue:ti,ab,kw OR anxiet*:ti,ab,kw OR depress*:ti,ab,kw OR hrqol:ti,ab,kw OR 'health related quality of life':ti,ab,kw OR 'life quality':ti,ab,kw OR 'return to work':ti,ab,kw OR 'back to work':ti,ab,kw OR memory:ti,ab,kw OR speech:ti,ab,kw OR aphasia:ti,ab,kw OR communicat*:ti,ab,kw OR 'social limit*':ti,ab,kw OR 'social participat*':ti,ab,kw OR 'social engage*':ti,ab,kw OR 'quality of life':ti,ab,kw OR impairment*:ti,ab,kw	3189204
#5	'cognition'/exp OR 'cognitive defect'/exp OR 'fatigue'/exp OR 'anxiety'/exp OR 'depression'/exp OR 'major depression'/exp OR 'return to work'/exp OR 'speech disorder'/exp OR 'communication disorder'/exp OR 'social participation'/exp OR 'quality of life'/exp	4581246
#4	#1 OR #2 OR #3	83228
#3	tia:ti,ab,kw	21913
#2	((minor OR mild OR mini OR 'non disabling' OR nondisabling OR transient OR 'non severe*' OR nonsevere* OR reversib*) NEAR/2 (stroke OR ischemi* OR ischaemi* OR cerebral OR cerebro* OR cva)):ti,ab,kw	52135
#1	'transient ischemic attack'/exp	46513

Cinahl

#	Query	Limiters/Expanders	Results
S9	S4 AND S7	Limiters - Published Date: 20130101-20231231<br>Search modes - Boolean/Phrase	**1,248**
S8	S4 AND S7	Search modes - Boolean/Phrase	2,049
S7	S5 OR S6	Search modes - Boolean/Phrase	1,074,878
S6	Cogniti* OR fatigue OR anxiet* OR Depress* OR HRQOL OR "Health Related Quality Of Life" OR "Life Quality" OR "return to work" OR "back to work" OR memory OR speech OR aphasia OR	Search modes - Boolean/Phrase	1,053,356

	communicat* OR "social limit*" OR "social participat*" OR "social engage*" OR "quality of life" OR impairment*		
S5	(MH "Fatigue+") OR (MH "Cognition+") OR (MH "Cognition Disorders+") OR (MH "Social Cognition") OR (MH "Anxiety+") OR (MH "Depression+") OR (MH "Job Re-Entry") OR (MH "Speech Disorders+") OR (MH "Social Participation") OR (MH "Quality of Life+")	Search modes - Boolean/Phrase	441,685
S4	S1 OR S2 OR S3	Search modes - Boolean/Phrase	12,838
S3	tia	Search modes - Boolean/Phrase	3,614
S2	(minor OR mild OR mini OR "non disabling" OR nondisabling OR transient OR "non severe*" OR nonsevere* OR reversib*) N2 (stroke OR ischemi* OR ischaemi* OR cerebral OR cerebro* OR CVA)	Search modes - Boolean/Phrase	11,632
S1	(MH "Cerebral Ischemia, Transient")	Search modes - Boolean/Phrase	5,211

Cochrane Library

ID	Search	Hits
#1	MeSH descriptor: [Ischemic Attack, Transient] explode all trees	950
#2	((minor OR mild OR mini OR "non disabling" OR nondisabling OR transient OR "non severe*" OR nonsevere* OR reversib*) NEAR/2 (stroke OR ischemi* OR ischaemi* OR cerebral OR cerebro* OR CVA)):ti,ab,kw	4823
#3	(TIA):ti,ab,kw	2037
#4	#1 OR #2 OR #3	5597
#5	MeSH descriptor: [Cognition] explode all trees	13537
#6	MeSH descriptor: [Cognition Disorders] explode all trees	7010
#7	MeSH descriptor: [Fatigue] explode all trees	4898
#8	MeSH descriptor: [Anxiety] explode all trees	10443
#9	MeSH descriptor: [Depression] explode all trees	15880
#10	MeSH descriptor: [Depressive Disorder] explode all trees	14894
#11	MeSH descriptor: [Return to Work] explode all trees	310
#12	MeSH descriptor: [Speech Disorders] explode all trees	1095

#13	MeSH descriptor: [Social Communication Disorder] explode all trees	15
#14	MeSH descriptor: [Social Participation] explode all trees	163
#15	MeSH descriptor: [Quality of Life] explode all trees	35073
#16	(Cogniti* OR fatigue OR anxiet* OR Depress* OR HRQOL OR "Health Related Quality Of Life" OR "Life Quality" OR "return to work" OR "back to work" OR memory OR speech OR aphasia OR communicat* OR "social limit*" OR "social participat*" OR "social engage*" OR "quality of life" OR impariment*):ti,ab,kw	369929
#17	#5 OR #6 OR #7 OR #8 OR #9 OR #10 OR #11 OR #12 OR #13 OR #14 OR #15 OR #16	372126
#18	#4 AND #17 with Cochrane Library publication date Between Jan 2013 and Feb 2023, in Cochrane Reviews, Trials	**881** 12 Reviews, **869 ials.**

PsycInfo

547 Results for

(((Year: [2013 TO 2023] OR TestYear: [2013 TO 2023]))) AND ((((((IndexTermsFilt: ("Cognitive Impairment"))) OR ((IndexTermsFilt: ("Cognition"))) OR ((IndexTermsFilt: ("Fatigue"))) OR ((IndexTermsFilt: ("Anxiety"))) OR ((IndexTermsFilt: ("Anxiety Sensitivity"))) OR ((IndexTermsFilt: ("Climate Anxiety"))) OR ((IndexTermsFilt: ("Computer Anxiety"))) OR ((IndexTermsFilt: ("Death Anxiety"))) OR ((IndexTermsFilt: ("Health Anxiety"))) OR ((IndexTermsFilt: ("Mathematics Anxiety"))) OR ((IndexTermsFilt: ("Performance Anxiety"))) OR ((IndexTermsFilt: ("Social Anxiety"))) OR ((IndexTermsFilt: ("Speech Anxiety"))) OR ((IndexTermsFilt: ("Test Anxiety"))) OR ((IndexTermsFilt: ("Travel Anxiety"))) OR ((IndexTermsFilt: ("Depression (Emotion)"))) OR ((IndexTermsFilt: ("Major Depression"))) OR ((IndexTermsFilt: ("Reemployment"))) OR ((IndexTermsFilt: ("Speech Disorders"))) OR ((IndexTermsFilt: ("Aphasia"))) OR ((IndexTermsFilt: ("Articulation Disorders"))) OR ((IndexTermsFilt: ("Dysphonia"))) OR ((IndexTermsFilt: ("Echolalia"))) OR ((IndexTermsFilt: ("Mutism"))) OR ((IndexTermsFilt: ("Stuttering"))) OR ((IndexTermsFilt: ("Social Communication"))) OR ((IndexTermsFilt: ("Social Functioning"))) OR ((IndexTermsFilt: ("Social Skills"))) OR ((IndexTermsFilt: ("Quality of Life"))) OR ((IndexTermsFilt: ("Health Related Quality of Life"))) OR ((IndexTermsFilt: ("Quality of Work Life"))))) OR ((((title: (Cogniti*))) OR ((title: (fatigue))) OR ((title: (anxiet*))) OR ((title: (Depress*))) OR ((title: (HRQOL))) OR ((title: ("Health Related Quality Of Life"))) OR ((title: ("Life Quality"))) OR ((title: ("return to work"))) OR ((title: ("back to work"))) OR ((title: (memory))) OR ((title: (speech))) OR ((title: (aphasia))) OR ((title: (communicat*))) OR ((title: ("social limit*"))) OR ((title: ("social participat*"))) OR ((title: ("social engage*"))) OR ((title: ("quality of life"))) OR ((title: ("impairment*")))) OR (((abstract: (Cogniti*))) OR ((abstract: (fatigue))) OR ((abstract: (anxiet*))) OR ((abstract: (Depress*))) OR ((abstract: (HRQOL))) OR ((abstract: ("Health Related Quality Of Life"))) OR ((abstract: ("Life Quality"))) OR

((abstract: ("return to work"))) OR ((abstract: ("back to work"))) OR ((abstract: (memory))) OR ((abstract: (speech))) OR ((abstract: (aphasia))) OR ((abstract: (communicat*))) OR ((abstract: ("social limit*"))) OR ((abstract: ("social participat*"))) OR ((abstract: ("social engage*"))) OR ((abstract: ("quality of life"))) OR ((abstract: ("impairment*")))))) AND (((((title: (TIA)))) OR (((abstract: (TIA))))) OR ((((title: (minor))) OR ((title: (mild))) OR ((title: (mini))) OR ((title: ("non disabling"))) OR ((title: (nondisabling))) OR ((title: (transient))) OR ((title: ("non severe*"))) OR ((title: (nonsevere*))) OR ((title: (reversib*)))) NEAR/2 (((title: (stroke))) OR ((title: (ischemi*))) OR ((title: (ischaemi*))) OR ((title: (cerebral))) OR ((title: (cerebro*))) OR ((title: (CVA)))) OR (((abstract: (minor))) OR ((abstract: (mild))) OR ((abstract: (mini))) OR ((abstract: ("non disabling"))) OR ((abstract: (nondisabling))) OR ((abstract: (transient))) OR ((abstract: ("non severe*"))) OR ((abstract: (nonsevere*))) OR ((abstract: (reversib*)))) NEAR/2 (((abstract: (stroke))) OR ((abstract: (ischemi*))) OR ((abstract: (ischaemi*))) OR ((abstract: (cerebral))) OR ((abstract: (cerebro*))) OR ((abstract: (CVA)))))))
